# Burden of autism spectrum disorders in North Africa and Middle East from 1990 to 2019: A systematic analysis for the Global Burden of Disease Study 2019

**DOI:** 10.1002/brb3.3067

**Published:** 2023-06-22

**Authors:** Sepideh Ebrahimi Meimand, Zahra Amiri, Parnian Shobeiri, Mohammad‐Reza Malekpour, Sahar Saeedi Moghaddam, Ali Ghanbari, Yeganeh Sharifnejad Tehrani, Zahra Shokri Varniab, Ashkan Pourabhari Langroudi, Hanye Sohrabi, Elmira Foroutan Mehr, Negar Rezaei, Maziar Moradi‐Lakeh, Ali H. Mokdad, Bagher Larijani

**Affiliations:** ^1^ Non‐Communicable Diseases Research Center, Endocrinology and Metabolism Population Sciences Institute Tehran University of Medical Sciences Tehran Iran; ^2^ Kiel Institute for the World Economy Kiel Germany; ^3^ Preventive Medicine and Public Health Research Center, Psychosocial Health Research Institute Iran University of Medical Sciences Tehran Iran; ^4^ Institute for Health Metrics and Evaluation, University of Washington Seattle Washington United States; ^5^ Endocrinology and Metabolism Research Center, Endocrinology and Metabolism Clinical Sciences Institute Tehran University of Medical Sciences Tehran Iran; ^6^ Department of Health Metrics Sciences, School of Medicine University of Washington Seattle Washington United States

**Keywords:** developmental delay, developmental disability, disability‐adjusted life years, epidemiology

## Abstract

**Introduction:**

Autism spectrum disorders (ASD) encompass a range of neurodevelopmental disorders that affect the patient's communication and behavior. There are some reports about the increasing prevalence of ASD in recent decades, mostly due to the improvement in diagnosis and screening status. Few studies suggested a lower prevalence of ASD in North Africa and Middle East compared to more developed regions. The aim of this study is to provide a comprehensive outlook of ASD in the region.

**Methods:**

We used Global Burden of Disease (GBD) data from 1990 to 2019 in North Africa and Middle East, which is one of the seven super regions of the GBD categorization. In this study, we reported the epidemiologic indices, including prevalence, incidence, and years lived with disability (YLDs) for ASD in the 21 countries of the super region. We also compared these indices between the countries based on their sociodemographic index (SDI) which was calculated according to income per capita, mean education, and fertility rate.

**Results:**

Age‐standardized prevalence rate (ASPR) of ASD in the region is 304.4 (95% uncertainty interval 251.2–366.1) per 100,000 in 2019 with less than one percentage change since 1990. Age‐standardized YLDs and incidence rates were 46.4 (30.4–67.5) and 7.7 (6.3–9.3) per 100,000 in 2019. The ASPR was 2.9 times greater in males compared to females in 2019. The highest age‐standardized prevalence, incidence, and YLD rates among the countries were seen in Iran in 2019 (370.3, 9.3, and 56.4 per 100,000, respectively). High SDI countries had higher age‐standardized YLDs rates compared to the other countries of the region.

**Conclusion:**

In conclusion, the trends of age‐standardized epidemiologic indices remained approximately steady through the years 1990–2019 in the region. Though, there was a wide discrepancy between the countries of the region. The difference of YLDs among the countries of this region is related to the SDI of the countries. Monetary and public awareness status are the SDI factors that may affect the quality of life of ASD patients in the region. This study provides valuable information for governments and health systems to implement policies for maintaining the improving trend, achieving more timely diagnosis, and bettering the supportive actions in this region.

## Graphical abstract

In this paper, we provided a prospect for health officials of the countries to implement their health care policies and programs and we also mentioned the possible ways to enhance the quality of life for these patients.

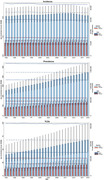

## 1 INTRODUCTION

Autism spectrum disorders (ASD) are defined as a wide range of neurodevelopmental disorders, including autism, pervasive developmental disorder‐not otherwise specified (PDD‐NOS), and Asperger's syndrome. According to the Diagnostic and Statistical Manual of Mental Disorders, 5th Edition (DSM‐5), the main features of ASD are persistent social‐communication deficits, restricted interests, and repetitive patterns of behavior. The symptoms begin in early childhood and generally last throughout a person's life (American Psychiatric Association, 2013). It can be sometimes diagnosed at 18 months or earlier (Hyman et al, 2020). There has been a dramatic increase in the prevalence of ASD in recent decades globally (Fombonne, 2001, 2020). Previously, ASD was considered to occur mostly in Western countries which have high technologies (Sanua, 1984). The Centers for Disease Control and Prevention (CDC) has been tracking the data about ASD in the United States since 1996; the estimated prevalence of ASD in 2000 was 6.7 per 1000 and it had raised to 16.8 per 1000 in 2014 and 18.5 per 1000 in 2016 (Autism and Developmental Disabilities Monitoring Network Surveillance Year 2000 Principal Investigators; Centers for Disease Control and Prevention, 2007; Autism and Developmental Disabilities Monitoring Network Surveillance Year 2002 Principal Investigators; Centers for Disease Control and Prevention, 2007; Baio et al., 2018; Rice et al., 2010; Van Naarden Braun et al., 2015). Over the last two decades, the increase in prevalence and knowledge of ASD has been documented in other parts of the world as well (Fombonne, 2003). However, there is no study about the epidemiology of ASD specifically addressed to North Africa and Middle East to date.

ASD happens in all ethnic, regional, racial, and socioeconomic groups, but the prevalence is varied among them, which might be because of dissimilar diagnoses among these groups. ASD is more prevalent in Caucasian children than Black or Hispanic children in the United States (Baio et al., 2018). The diversity seems to diminish, but the still existing discrepancy can be the outcome of stigma and varied accessibility of health services (Hodges et al., 2020). Gender has a determined impact on the prevalence of ASD as if ASD is more prevalent in males compared to females, previously reported 4:1 ratio (Fombonne, 2009). Recently, a meta‐analysis reported a ratio of 3:1 (Loomes et al., 2017). Some studies suggest that females are more likely to be overlooked, misdiagnosed, or diagnosed lately (Bargiela et al., 2016; Giarelli et al., 2010; Russell et al., 2011).

The majority of North Africa and Middle East countries are considered developing and less developed (Prates & Baltar, 2020; Santos & Santos, 2014). The prevalence of ASD in less developed countries is lower compared to Western countries; it may be a result of stigmatization in these countries and the tendency of parents to send their children to general education schools rather than special schools (Baird et al., 2006; Samadi, 2008). Another reason might be the deprivation of children with intellectual disabilities from educational services at an early age and the screening before enrolling in elementary schools (Hosseinpoor et al., 2005). It seems more probable that the lower prevalence is due to a lack of diagnostic resources and other external factors. Few studies have reported the epidemiology of ASD in North Africa and Middle East. Overall, these reports suggest a lower prevalence of ASD in the Middle East compared to the Western countries (Chiarotti & Venerosi, 2020).

This report provides the latest available estimates about the prevalence, incidence, and years lived with disability (YLDs) of ASD in North Africa and Middle East nationally. It also describes the evolution of ASD epidemiology in this area from 1990 to 2019.

## 2 MATERIALS AND METHODS

ASD was defined as a group of five sub‐disorders: Autistic disorder (299), PDD, PDD‐NOS (299.80), Rett's disorder (299.8), Asperger's disorder (299.8), and childhood disintegrative disorder (299.10) according to the Diagnostic and Statistical Manual of Mental Disorders (fourth edition, text revision DSM‐IV‐TR) (Cooper, 2000). But, it had been incorporated into a single disorder in the DSM‐5 (American Psychiatric Association, 2013).

The Global Burden of Disease (GBD) inclusion criteria of studies used for estimations, described in detail, are available elsewhere (GBD 2019 Diseases and Injuries Collaborators, 2020).

### 2.1 Data input

In this analysis, we have collected data from a systematic review done for the Global Burden of Diseases, Injuries, and Risk Factors Study (GBD) 2019. GBD 2019 cumulated a large number of input sources to estimate mortality, causes of death and illness, and risk factors for 369 conditions in 204 countries and territories from 1990 to 2019. The North Africa and Middle East, which is one of the 7 super regions and one of the 21 regions of the GBD categorization, includes 21 countries: Afghanistan, Algeria, Bahrain, Egypt, Iran, Iraq, Jordan, Kuwait, Lebanon, Libya, Morocco, Oman, Palestine, Qatar, Saudi Arabia, Sudan, Syrian Arabic Republic, Tunisia, Turkey, United Arab Emirates, and Yemen. The GBD study provides a standardized analytical approach for modeling and estimating incidence, prevalence, and YLDs by age, sex, cause, year, and location. Data sources for models are available online at the Global Health Data Exchange website (Institute for Health Metrics and Evaluation).

### 2.2 Nonfatal estimates

Epidemiological outcomes for ASD were modeled using DisMod‐MR 2.1, a Bayesian meta‐regression modeling tool developed for GBD epidemiological analyses. A meta‐regression with Bayesian priors, regularization, and trimming (MR‐BRT) analysis was used to run a nested network meta‐regression to estimate adjustments to alternative data prior to running DisMod‐MR 2.1. In addition, MR‐BRT was used to run a meta‐regression analysis on the within‐study sex ratios to estimate a pooled sex ratio with a 95% uncertainty interval (UI). UI is defined as a range of values that shows the certainty of an estimate (GBD 2016 Neurology Collaborators, 2019) and was generated for every metric using the 25th and 975th ordered 1000 draw values of the posterior distribution. In this study, we reported each incidence, prevalence, and YLD estimates with its 95% UI.

We aimed to report the epidemiological status of ASD in terms of annual prevalence, incidence, and disability‐adjusted life‐years (DALYs) by age, sex, and country from 1990 to 2019 (GBD 2019 Diseases and Injuries Collaborators, 2020). Calculating the age‐standardized rates was accomplished using the GBD standard population in this study. DALYs are calculated as the sum of the years of life lost due to premature mortality and the YLDs for people living with the health condition. In this study, we did not estimate premature mortality due to ASD; because medical literature maintained that ASD does not affect life expectancy directly and GBD does not report any deaths from ASD as the underlying cause. As a result, DALYs for ASD are equivalent to YLDs (Baxter et al., 2014). As explained in the GBD 2019 Data Input Sources Tool, nine scientific literature sources have been used as inputs, which were directly related to North Africa and Middle East.

### 2.3 Sociodemographic index

GBD uses a compound measure of lag distributed income per capita, mean education for those ages 15 and older, and fertility rate under the age of 25 for all areas in the GBD study to define sociodemographic influence on health outcomes. Sociodemographic index (SDI) is expressed in five levels: high SDI, high–middle SDI, middle SDI, low‐middle SDI, and low SDI. We determined the ASD annual prevalence, incidence, and YLDs in all SDI quintiles using the GBD Results Tool. SDI in the region ranged from 0.34 in Afghanistan to 0.88 in the United Arab Emirates in 2019.

### 2.4 Health access and quality index

Health access and quality (HAQ) index provides estimations for the personal health care access and the quality of each location by using 32 causes leading to death that would be preventable in the presence of high‐quality health care access. In other words, it predicts amenable mortality. Principal components analysis was used to construct this index on the basis of mortality‐to‐incidence ratios for cancers and risk‐standardization of death rates for non‐cancer causes. HAQ index in the region ranged from 26.91 in Afghanistan to 82.52 in Kuwait in 2019.

### 2.5 Role of the funding source

The funder of the study had no role in study design, data collection, data analysis, data interpretation, or writing of the report. All authors had full access to the data in the study and had final responsibility for the decision to submit for publication.

## 3 RESULTS

### 3.1 Prevalence

The number of prevalent cases of ASD in North Africa and Middle East was 1105,582 (911,505–1326,857) in 1990 and increased by 70.0% (68.7–71.5) to 1879,528 (1550,850–2261,649) in 2019. It forms 6.6% of the whole world's cases of ASD in 2019 (Table S1). Age‐standardized prevalence rate (ASPR) per 100,000 in males (446.6; 369.4–536.3) was 2.9 times greater than females (151.4; 122.3–184.1) in 2019 (Table 1).

**TABLE 1:** All ages number and age‐standardized rate (per 100,000) of burden due to autism spectrum disorders (ASD) by sex in 1990 and 2019 with percent change, region

			Year						% Change (1990–2019)		
			1990			2019					
Location	Measure	Age, metric	Both	Female	Male	Both	Female	Male	Both	Female	Male
Global	Incidence	All ages (number)	602,887 (501,382 to 718,288)	141,254 (113,407–170,938)	461,633 (386,362–548,162)	603,790 (501,680–720,097)	144,297 (115,510–174,371)	459,493 (384,472–544,406)	0.1 (−0.2 to 0.5)	2.2 (1.7–2.6)	−0.5 (−0.9 to −0.1)
		Age‐standardized rate (per 100,000)	9.2 (7.6–10.9)	4.4 (3.6–5.4)	13.6 (11.4–16.1)	9.3 (7.7–11.1)	4.6 (3.7–5.6)	13.7 (11.5–16.3)	1.7 (1.4–2)	3.9 (3.4–4.4)	1 (0.6–1.3)
	Prevalence	All ages (number)	2033,6256 (16,857,367–24,222,582)	4688,533 (3797,052–5711,864)	15,647,724 (12,986,732–18,607,125)	28,324,939 (23,500,644–33,811,271)	6691,163 (5436,262–8153,529)	21,633,776 (17,978,516–25,761,348)	39.3 (38.6–40)	42.7 (41.9–43.5)	38.3 (37.5–39)
		Age‐standardized rate (per 100,000)	372.8 (309.1–444.9)	173.4 (140.9–211.5)	571.2 (473.8–679.6)	369.4 (305.9–441.2)	176.3 (143–214.5)	560.1 (465.2–667.3)	−0.9 (−1.3 to −0.6)	1.7 (1.3–2.1)	−1.9 (−2.3 to −1.6)
	YLDs	All ages (number)	3105,909 (2025,303–4514,467)	712,135 (467,655–1041,370)	2393,774 (1560,490–3443,926)	4306,615 (2821,512–6232,360)	1012,148 (663,237–1477,450)	3294,468 (2152,733–4769,103)	38.7 (37.7–39.7)	42.1 (40.4–43.9)	37.6 (36.6–38.7)
		Age‐standardized rate (per 100,000)	56.7 (37–82.2)	26.2 (17.2–38.2)	86.9 (56.7–125.1)	56.3 (36.8–81.5)	26.7 (17.5–39.2)	85.3 (55.8–123.6)	−0.8 (−1.4 to −0.2)	2 (0.9–3.1)	−1.8 (−2.4 to −1.1)
North Africa and Middle East	Incidence	All ages (number)	44,221 (36,225–53,111)	10,846 (8645–13,164)	33,375 (27,695–39,933)	45,002 (36,857–53,912)	11,041 (8776–13,429)	33,961 (28,098–40,476)	1.8 (1.0–2.5)	1.8 (0.9–2.7)	1.8 (0.9–2.7)
		Age‐standardized rate (per 100,000)	7.9 (6.5–9.5)	4.0 (3.2–4.8)	11.6 (9.6–13.9)	7.7 (6.3–9.3)	3.9 (3.1–4.7)	11.4 (9.4–13.5)	−1.9 (−2.6 to −1.2)	−1.9 (−2.7 to −1.0)	−1.9 (−2.8 to −1.0)
	Prevalence	All ages (number)	1105,582 (911,505–1326,857)	269,943 (217,347–327,533)	835,638 (691,702–1002,508)	1879,528 (1550,850–2261,649)	449,188 (362,609–545,371)	1430,340 (1184,252–1717,531)	70 (68.7–71.5)	66.4 (65–67.9)	71.2 (69.6–72.9)
		Age‐standardized rate (per 100,000)	303.5 (250.4–365.5)	151.7 (122.4–184.5)	448.4 (371.1–539.1)	304.4 (251.2–366.1)	151.4 (122.3–184.1)	446.6 (369.4–536.3)	0.3 (−0.3 to 0.9)	−0.2 (−0.9 to 0.5)	−0.4 (−1.2 to 0.4)
	YLDs	All ages (number)	170,028 (110,895–247,697)	41,335 (27,001–59,662)	128,693 (83,738–187,924)	287,616 (188,299–418,662)	68,403 (44,500–99,267)	219,214 (144,028–318,230)	69.2 (65.5–73.1)	65.5 (58.9–71.7)	70.3 (65.9–74.7)
		Age‐standardized rate (per 100,000)	46.3 (30.2–67.6)	23.0 (15.1–33.2)	68.5 (44.8–99.8)	46.4 (30.4–67.5)	23.0 (15.0–33.4)	68.2 (44.7–99.2)	0.4 (−1.8 to 2.4)	−0.1 (−3.7 to 3.7)	−0.4 (−2.8 to 2.0)

*Note*: Data in parenthesis are 95% uncertainty interval (95% UI).

Abbreviation: YLDs, years lived with disability.

**FIGURE 1:**
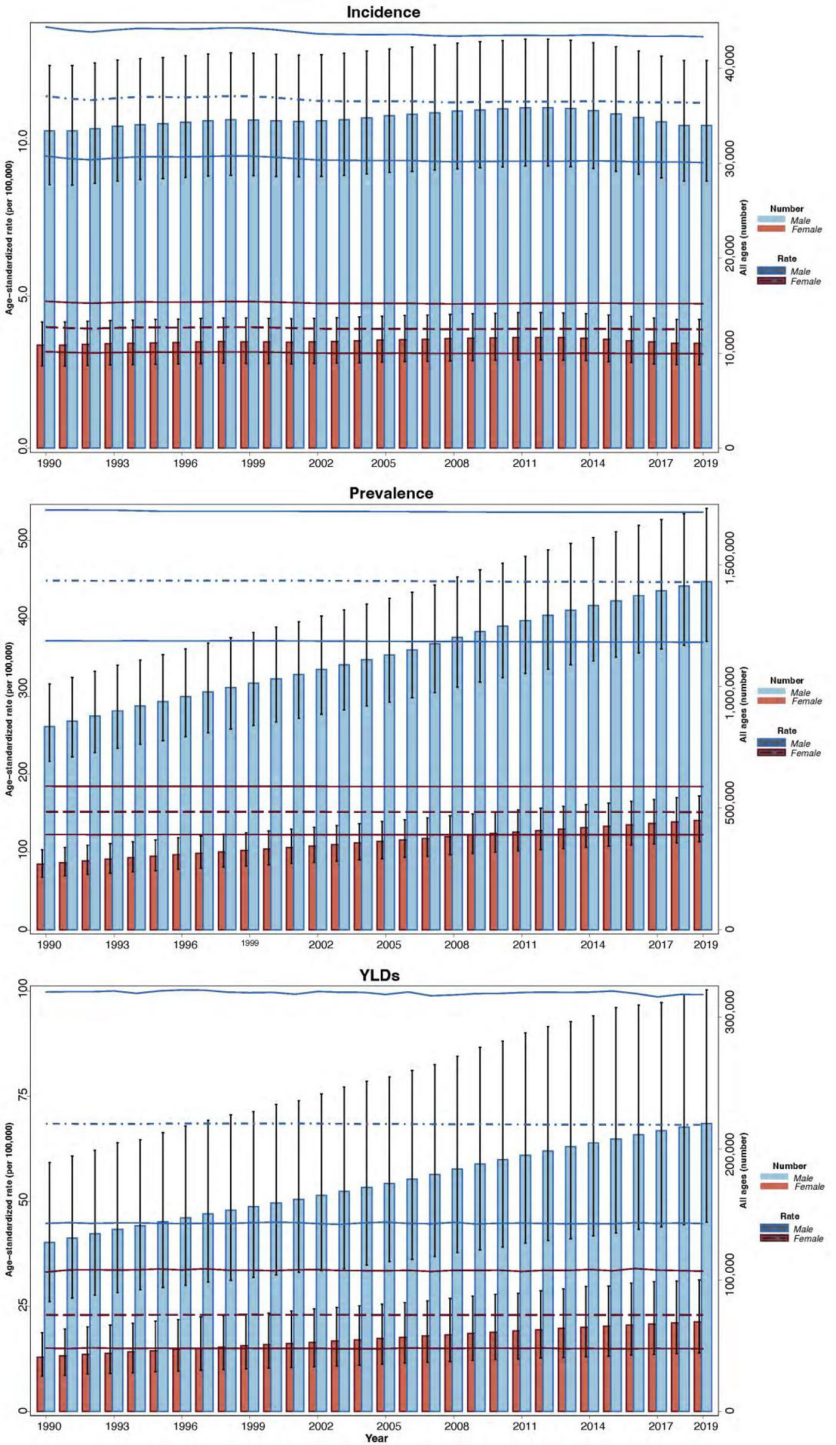
All ages number and age‐standardized rate of incidence, prevalence, and years lived with disability (YLDs) for autism spectrum disorders (ASD) by sex, 1990–2019, and 95% uncertainty interval.

**FIGURE 2:**
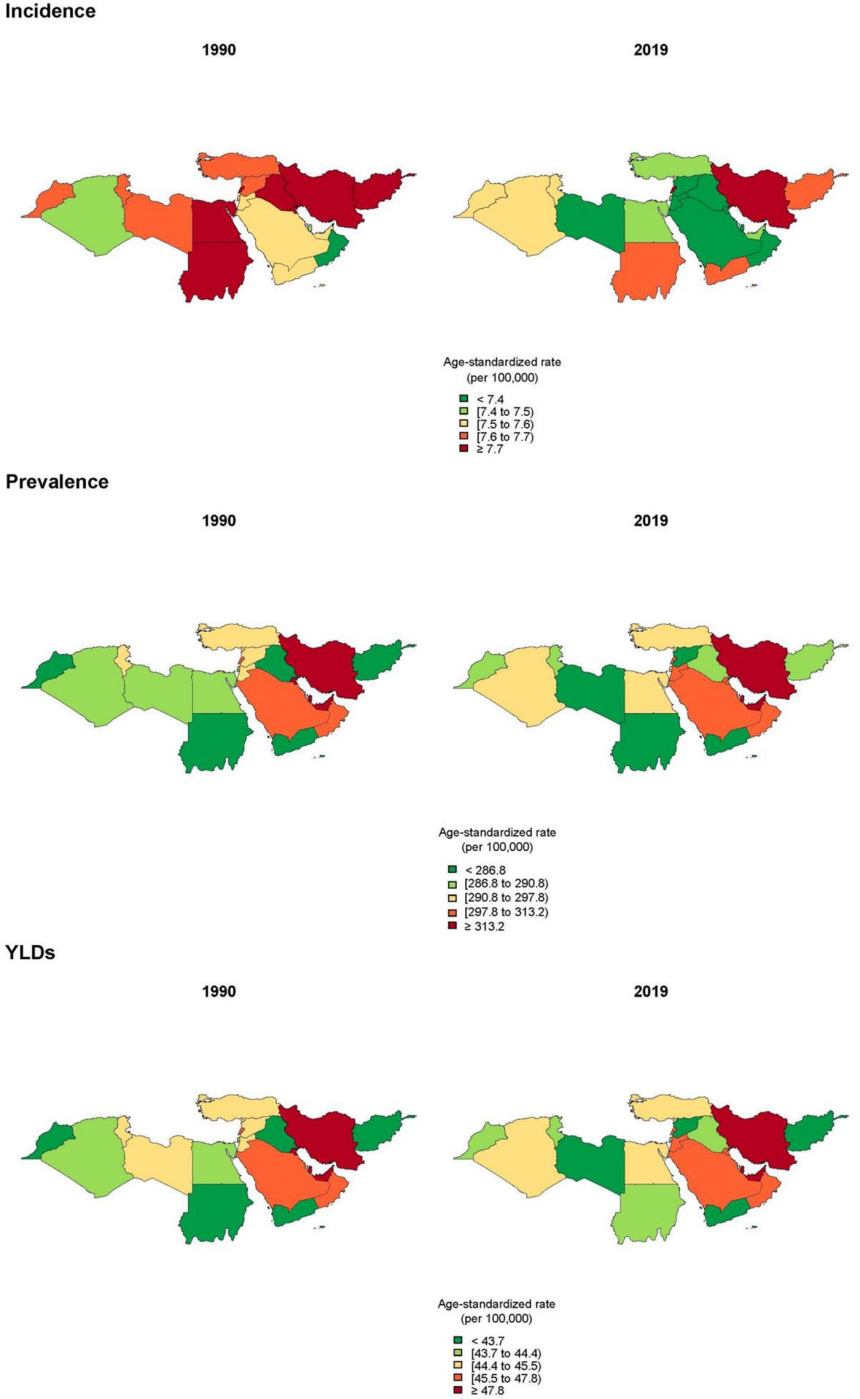
Comparison of age‐standardized rate of incidence, prevalence, and years lived with disability (YLDs) among North Africa and Middle East countries in 1990 and 2019.

The ASPR in North Africa and Middle East was 303.6 (250.4–365.5) and 304.4 (251.2–366.1) per 100,000 in 1990 and 2019, respectively (Figure 1). The ASPRs of ASD were the highest in Iran, Qatar, United Arab Emirates; 370.3 (306.8–441.4), 344.7 (283.2–415.7), and 331.2 (271.9–400.8) in 2019. The ASPRs of ASD were the lowest in the Syrian Arab Republic, Yemen, and Libya; 283.9 (231.3–345.4), 286.0 (234.6–347.0), and 286.5 (235.2–347.9) in 2019 (Table S1, Figure 2). The interquartile range (IQR) of ASPR in the region declined from 1990 to 2019 (21.4 to 17.9 per 100,000 population).

ASPRs have been higher in countries with higher SDI among North Africa and Middle East countries compared to the countries with lower SDI.

### 3.2 Incidence

The number of new ASD cases in North Africa and Middle East region has increased by 1.8% (1.0–2.5) from 44,221 (36,225–53,111) in 1990 to 45,002 (36,857–53,912) in 2019 (Table 1). It forms 7.5% of the whole world's new cases of ASD in 2019. Age‐standardized incidence rate (ASIR) in males (11.4; 9.4–13.5) was 2.9 times higher than in females (3.9; 3.1–4.7) in 2019 (Table 1).

The ASIRs in North Africa and Middle East were 7.9 (6.5–9.5) and 7.7 (6.3–9.3) per 100,000 in 1990 and 2019, respectively (Figure 1). ASIRs of ASD were estimated to be the highest in Iran, Lebanon, and Sudan; 9.3 (7.7–11.1), 7.9 (6.4–9.5), and 7.7 (6.3–9.3) in 2019, respectively. ASIRs of ASD were estimated to be the lowest in Oman, Qatar, and Jordan; 7.1 (5.8–8.5), 7.3 (5.9–8.7), and 7.3 (5.9–8.8) in 2019, respectively (Table S1, Figure 2). The IQR of ASIR in the region declined from 1990 to 2019 (0.24 to 0.16 per 100,000 population).

### 3.3 YLDs/DALYs

The numbers of YLDs and DALYs are the same because of the nonfatal nature of ASD. Although their absolute numbers are the same, the share of ASD from total YLDs and total DALYs is different. The all‐ages YLDs of ASD in North Africa and Middle East comprised 6.7% of total YLDs in 2019, and the share of DALYs in the region is 0.17% of the total. The all‐ages YLDs rate of ASD in North Africa and Middle East has decreased by 4.1% (−6.2 to −1.9) from 49.3 (32.1–71.8) in 1990 to 47.25 (30.9–68.8) in 2019. Age‐standardized YLDs rate (ASYR) in males (68.2; 44.7–99.2) was three times greater than females (23.0; 15.0–33.4) in 2019 (Table 1).

The ASYR of ASD in North Africa and Middle East was 46.3 (30.2–67.6) and 46.4 (30.4–67.5) per 100,000 in 1990 and 2019, respectively (Figure 1). ASYR of the region was the second lowest amongst all the super regions through 1990–2019. The ASYRs in 2019 were the highest in Iran, Qatar, and United Arab Emirates; 56.4 (37.0–81.7), 52.6 (34.0–75.9), and 50.5 (33.0–73.5) in 2019, respectively. The ASYRs in 2019 were the lowest in the Syrian Arab Republic, Afghanistan, and Yemen; 43.2 (28.6–63.4), 43.4 (28.6–62.8), and 43.5 (28.3–62.8) in 2019, respectively (Figure 2). The IQR of ASYR in the region declined from 1990 to 2019 (3.4 to 2.8 per 100,000 population).

**FIGURE 3:**
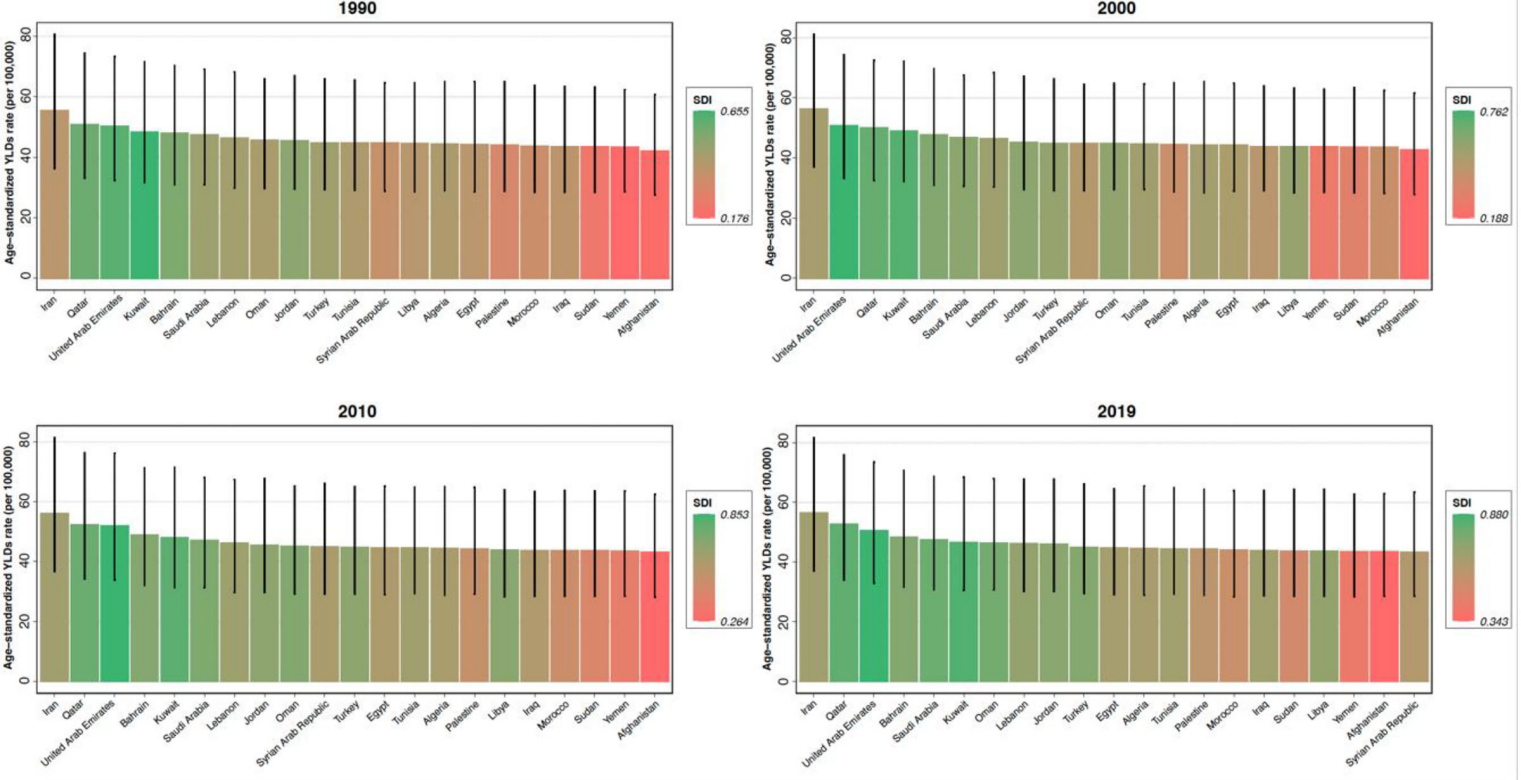
Age‐standardized years lived with disability (YLDs) rate for autism spectrum disorders (ASD) among North Africa and Middle East countries by sociodemographic index (SDI), 1990–2019 and 95% uncertainty intervals.

ASYR has been higher in countries with higher SDI among the region's countries. Figure 3 shows the consistency of these results from 1990 to 2019 within a 10‐year interval.

The YLDs number of prevalent cases was used as an indicator of the personal cost of illness for each patient. This ratio is 0.153 YLD per patient for ASD compared to 0.108 YLD per patient for all causes in 2019 in the region.

## 4 DISCUSSION

Our study revealed a high epidemiologic rate in the region with a large variation between countries by SDI and HAQ. The epidemiology of ASD has not been well studied in the region, and more efforts are needed to accurately assess the burden and address it. Our results are of great value as they show the epidemiological status of disease by country and allow health officials and others to design and implement programs to address ASD and monitor progress using future GBD estimates.

Iran, Qatar, and United Arab Emirates had the highest all‐ages rates and ASPR of ASD in the region. These countries’ HAQ indices were above the average of the region, which indicates that their populations had better access to health care facilities. Thus, higher reported prevalence rates in these countries may reflect better diagnosis and it does not prove the higher prevalence of ASD in these countries necessarily. Furthermore, these countries are considered to have better sociodemographic status among the countries of the region. It suggests there may be a correlation between ASD prevalence and SDI components, including education, income, and fertility. A study reported that physicians, teachers, and psychiatrists are those who refer patients for clinical assessment the most among health and education professionals (Albores‐Gallo & Solís‐Bravo, 2019), which explains the relationship between higher education and income with higher prevalence. Moreover, human and physical resources facilitate identification and diagnosis, which are more accessible in high‐income countries (Samms‐Vaughan, 2014). The decline of the IQR in our measures shows that the countries of the region have become more equal in ASD prevalence, incidence, and cost of illness in recent years.

The annual incidence of ASD in North Africa and Middle East has increased between the years 1990–2019, whereas the ASIR has decreased insignificantly and remained steady. The incremental trend seen for incidence may be due to population growth. Nevertheless, changes in ASIR over these years were insignificant. ASD can be diagnosed at 18 months or earlier; the sooner diagnosis happens, the better outcomes of the treatment would be. Maureen et al. reported that in middle and low‐income countries the mean age at diagnosis ranges between 45 and 57 months (Samms‐Vaughan, 2014). One study in 2005 reported the mean age at diagnosis in Pennsylvania, United States of America (USA) is approximately 37 months (Mandell et al., 2005), another study stated the mean age is about 38 months in the USA (Rosenberg et al., 2011). Samms‐Vaughan and Franklyn‐Banton (2008) reported Low maternal education as a risk factor for delayed diagnosis.

The ASYR of ASD has increased by 0.37% from 1990 to 2019 in North Africa and Middle East, whereas the global YLDs have decreased by 0.75%. The gradual increasing trend in this region may be due to improved public awareness, health care accessibility, screening, and diagnostic substitution (Shattuck, 2006). The diagnostic substitution refers to the change that many children are now being diagnosed with ASD, whereas they were diagnosed with mental retardation and learning disabilities previously (Shattuck, 2006).

DSM‐5 was published in 2013, and DSM‐IV‐TR was used for ASD diagnosis till 2013. A study in 2014 reviewed the effect of DSM‐5 on the epidemiology of ASD; 9%–54% of cases diagnosed with DSM‐IV do not qualify for DSM‐5 (Tsai, 2014). This suggests the estimated number of new cases in recent years could be more if the old version of DSM was used. GBD used a crosswalk approach to adjust the estimate based on the DSM‐5 criteria as the reference definition.

The prevalence, incidence, and DALY of ASD were higher in males compared to females in North Africa and Middle East same as global estimations. The previous reports of male‐to‐female ratios vary between 4:1 and 3:1 globally (Fombonne, 2009; Loomes et al., 2017). Some reasons explain the ratio: (1) female autism phenotype; presented with less overt restricted interests, (2) females’ tendency to mask their autistic deficits through a process known as “camouflaging,” and (3) professionals, who play a role in diagnosis (family physicians, teachers, pediatricians, psychologists, psychiatrists, etc.), assuming ASD as a male disorder (Bargiela et al., 2016; Frazier et al., 2014; Mersch et al., 2015).

There are several factors affecting timely diagnosis and the quality of life in ASD patients. Some of these factors are known to reduce the delayed diagnosis of ASD such as increasing public awareness and promoting primary care established on a surveillance and screening basis. Furthermore, training of early detection of ASD for general physicians, preschool educators, and other professionals who have contact with children at early ages may lead to early diagnosis (Masri et al., 2013; Samms‐Vaughan & Franklyn‐Banton, 2008). Even in the presence of an accurate diagnosis, ASD patients and their families need medical support and facilities to help them live with their condition. These are some qualities that are absent in many countries of this region. In this study, we showed that countries with lower HAQ index and SDI require more endeavor to equip folks, train medical personnel, establish more clinics, and implement their databases.

We used GBD data for this study which is based on regional and global reports and papers, so the numbers are affected by the research done in each country. Availability and access to high‐quality data is a major issue in the North Africa and Middle East. There has been progress in the diagnosis ability throughout the years, which we were not able to consider in the analysis. The discrepancy in health care availability and approaches among different countries of the region is another factor that could affect the results; however, it was not doable to completely investigate the effects in the analysis. For mental disorders, there might be more limitations because of the stigma. Far more than coping with the destructive effects of the mental disorder, these patients have to face the difficulty of becoming excluded from social groups and prejudices (Rössler, 2016). The incidence of ASD was not available by age group, so we were unable to evaluate the age of diagnosis and screening status in different countries of the region.

## 5 CONCLUSION

In conclusion, ASD is an important cause of disability starting from early childhood and lasting through a lifetime. Since most of our knowledge of ASD comes from high‐income countries, there is an urge to fill in the knowledge gap. The prevalence and incidence of ASD have increased in North Africa and Middle East; population growth, improved public awareness, early detection, and promotion of primary care may be the reasons behind the changes. Regarding the variation seen in the quality of accessible health care in this region, the countries can focus on bettering their health care status to develop. This report provides a general overview of the epidemiologic status of ASD, using the GBD study 2019, for health care facilities, health policymakers, and researchers to achieve a timely diagnosis, early intervention, and better surveillance in this area.

## AUTHOR CONTRIBUTIONS

**Sepideh Ebrahimi Meimand, Zahra Amiri**: Conceptualization; writing–original draft; writing–review and editing. **Parnian Shobeiri**: Data curation; methodology; visualization. **Mohammad‐Reza Malekpour**: Conceptualization; formal analysis; writing–review and editing. **Sahar Saeedi Moghaddam**: Conceptualization; formal analysis; validation; visualization; writing–review and editing. **Ali Ghanbari**: Formal analysis; methodology. **Yeganeh Sharifnejad Tehrani**: Data curation; formal analysis; visualization. **Zahra Shokri Varniab, Ashkan Pourabhari Langroudi**: Validation; writing–review and editing. **Hanye Sohrabi**: Conceptualization; investigation. **Elmira Foroutan Mehr**: Project administration. **Negar Rezaei**: Conceptualization; supervision; validation. **Maziar Moradi‐Lakeh**: Conceptualization; investigation; supervision; validation; writing–review and editing. **Ali H. Mokdad, Bagher Larijani**: Supervision; validation; writing–review and editing

## CONFLICT OF INTEREST STATEMENT

None of the authors has any conflict of interest to declare.

### PEER REVIEW

The peer review history for this article is available at https://publons.com/publon/10.1002/brb3.3067.

## Supporting information

Table S1 Age‐standardized rate of incidence, prevalence, and YLDs for ASD in North Africa and Middle East countries in 1990 and 2019 with percent change.

## Data Availability

The data that support the findings of this study are openly available in GBD Results tool at [https://vizhub.healthdata.org/gbd‐results/].
