# Supplementary material for: Burden of autism spectrum disorders in North Africa and Middle East from 1990 to 2019: A systematic analysis for the Global Burden of Disease Study 2019
Source: Brain Behav. 2023 Jun 22;13(7):e3067. doi: 10.1002/brb3.3067 (PMC10338812; doi:10.1002/brb3.3067)
Supplement: Supplementary file 1 — Table S1 Age‐standardized rate of incidence, prevalence, and YLDs for ASD in North Africa and Middle East countries in 1990 and 2019 with percent change. [file BRB3-13-e3067-s001.docx]

**Supplementary table 1.** Age-standardized rate of incidence, prevalence, and YLDs for ASD in North Africa and Middle East countries in 1990 and 2019 with percent change

| Country | Measure | 1990 | 2019 | % Change (1990 to 2019) |
| --- | --- | --- | --- | --- |
| Afghanistan | Incidence | 7.7 (6.2 to 9.3) | 7.6 (6.2 to 9.2) | -0.9 (-0.9 to -0.9) |
|  | Prevalence | 280.9 (228.3 to 340.4) | 287.1 (233.2 to 347.9) | 2.2 (2 to 2.5) |
|  | YLDs | 42.1 (27.5 to 60.8) | 43.4 (28.6 to 62.8) | 3.2 (-3.8 to 10.6) |
| Algeria | Incidence | 7.5 (6.1 to 9) | 7.6 (6.2 to 9.1) | 1.4 (1.4 to 1.4) |
|  | Prevalence | 290 (237.6 to 349) | 290.9 (238.3 to 350) | 0.3 (0.3 to 0.3) |
|  | YLDs | 44.4 (28.9 to 65) | 44.5 (28.9 to 65.6) | 0.3 (-6.3 to 7.1) |
| Bahrain | Incidence | 7.5 (6.1 to 9) | 7.4 (6 to 9) | -0.4 (-0.4 to -0.4) |
|  | Prevalence | 313.8 (253.7 to 379.9) | 316.6 (255.9 to 382.9) | 0.9 (0.8 to 1) |
|  | YLDs | 47.9 (31 to 70.3) | 48.3 (31.7 to 70.8) | 0.9 (-6.2 to 8.1) |
| Egypt | Incidence | 7.8 (6.3 to 9.4) | 7.5 (6 to 9.1) | -3.9 (-3.9 to -3.9) |
|  | Prevalence | 289.5 (235.8 to 350.8) | 292.2 (238 to 353.9) | 0.9 (0.8 to 1) |
|  | YLDs | 44.2 (28.5 to 65) | 44.7 (29.1 to 64.6) | 1.1 (-5.9 to 7.8) |
| Iran | Incidence | 8.9 (7.4 to 10.7) | 9.3 (7.7 to 11.1) | 4.9 (2.6 to 7.5) |
|  | Prevalence | 364.3 (299.8 to 437.5) | 370.3 (306.8 to 441.4) | 1.6 (-0.9 to 4.4) |
|  | YLDs | 55.4 (36.2 to 80.6) | 56.4 (37 to 81.7) | 1.8 (-1.4 to 5.1) |
| Iraq | Incidence | 7.8 (6.3 to 9.4) | 7.4 (6 to 8.9) | -4.7 (-4.7 to -4.7) |
|  | Prevalence | 286.3 (231.5 to 345.3) | 286.8 (231.9 to 345.9) | 0.2 (0.2 to 0.2) |
|  | YLDs | 43.5 (28.4 to 63.3) | 43.8 (28.7 to 64) | 0.7 (-5.7 to 7.8) |
| Jordan | Incidence | 7.5 (6.1 to 9.1) | 7.3 (5.9 to 8.8) | -3.4 (-3.4 to -3.4) |
|  | Prevalence | 296.9 (243.5 to 360.9) | 299.9 (246 to 364.2) | 1 (0.9 to 1.1) |
|  | YLDs | 45.4 (29.5 to 67) | 46 (30.1 to 67.9) | 1.1 (-5.5 to 8.2) |
| Kuwait | Incidence | 7.7 (6.3 to 9.4) | 7.4 (6 to 8.9) | -4.7 (-4.7 to -4.6) |
|  | Prevalence | 315.9 (256.7 to 383.9) | 305 (248 to 370.1) | -3.5 (-3.8 to -3.1) |
|  | YLDs | 48.3 (31.5 to 71.6) | 46.6 (30.6 to 68.5) | -3.5 (-9.6 to 2.9) |
| Lebanon | Incidence | 8 (6.5 to 9.7) | 7.9 (6.4 to 9.5) | -1.9 (-1.9 to -1.9) |
|  | Prevalence | 303.7 (246.8 to 368.6) | 302.3 (245.6 to 367) | -0.5 (-0.5 to -0.4) |
|  | YLDs | 46.3 (29.8 to 68.2) | 46.2 (30.2 to 67.9) | -0.1 (-6.6 to 7.3) |
| Libya | Incidence | 7.6 (6.3 to 9.2) | 7.3 (6 to 8.8) | -4.2 (-4.2 to -4.2) |
|  | Prevalence | 290.8 (238.9 to 353.2) | 286.5 (235.2 to 347.9) | -1.5 (-1.7 to -1.3) |
|  | YLDs | 44.5 (28.6 to 64.6) | 43.7 (28.6 to 64.4) | -1.8 (-7.7 to 5.2) |
| Morocco | Incidence | 7.6 (6.2 to 9.2) | 7.6 (6.2 to 9.1) | -0.8 (-0.8 to -0.8) |
|  | Prevalence | 285.6 (234 to 344.9) | 287.5 (235.6 to 347.2) | 0.7 (0.6 to 0.7) |
|  | YLDs | 43.6 (28.4 to 63.7) | 43.9 (28.3 to 64) | 0.7 (-5.8 to 8.1) |
| Oman | Incidence | 7.1 (5.8 to 8.6) | 7.1 (5.8 to 8.5) | -0.7 (-0.7 to -0.7) |
|  | Prevalence | 298.4 (244.2 to 360.6) | 303 (248.2 to 366) | 1.5 (1.4 to 1.7) |
|  | YLDs | 45.6 (29.7 to 65.8) | 46.3 (30.6 to 67.9) | 1.6 (-5.5 to 9.4) |
| Palestine | Incidence | 7.5 (6.1 to 9.1) | 7.5 (6.1 to 9) | -0.7 (-0.7 to -0.7) |
|  | Prevalence | 288.6 (235.1 to 349.3) | 292.1 (237.8 to 353.6) | 1.2 (1.1 to 1.4) |
|  | YLDs | 44 (28.7 to 65) | 44.4 (28.9 to 64.4) | 1 (-5.8 to 8.3) |
| Qatar | Incidence | 7.5 (6.1 to 9) | 7.3 (5.9 to 8.7) | -2.9 (-2.9 to -2.9) |
|  | Prevalence | 332.5 (273.6 to 400.6) | 344.7 (283.2 to 415.7) | 3.7 (3.4 to 4) |
|  | YLDs | 50.8 (33 to 74.5) | 52.6 (34 to 75.9) | 3.6 (-3.5 to 10.9) |
| Saudi Arabia | Incidence | 7.5 (6.1 to 9.1) | 7.4 (6 to 9) | -0.7 (-0.7 to -0.7) |
|  | Prevalence | 310 (252.4 to 374.5) | 310.9 (253.2 to 375.6) | 0.3 (0.3 to 0.3) |
|  | YLDs | 47.3 (30.9 to 69.1) | 47.4 (30.8 to 68.7) | 0.1 (-6.5 to 7.1) |
| Sudan | Incidence | 8.1 (6.6 to 9.8) | 7.7 (6.3 to 9.3) | -5.5 (-5.5 to -5.5) |
|  | Prevalence | 285.4 (232.8 to 343) | 286.7 (233.9 to 344.7) | 0.5 (0.4 to 0.5) |
|  | YLDs | 43.4 (28.3 to 63.1) | 43.7 (28.6 to 64.3) | 0.7 (-6.4 to 8.2) |
| Syrian Arab Republic | Incidence | 7.7 (6.3 to 9.2) | 7.4 (6.1 to 8.9) | -3.2 (-3.2 to -3.2) |
|  | Prevalence | 292 (237.9 to 355.1) | 283.9 (231.3 to 345.4) | -2.8 (-3 to -2.5) |
|  | YLDs | 44.6 (28.8 to 64.6) | 43.2 (28.6 to 63.4) | -3.3 (-9.9 to 3.7) |
| Tunisia | Incidence | 7.6 (6.2 to 9.2) | 7.5 (6.1 to 9) | -1.5 (-1.6 to -1.5) |
|  | Prevalence | 291.8 (238.2 to 353.8) | 290.5 (237.2 to 352.2) | -0.5 (-0.5 to -0.4) |
|  | YLDs | 44.7 (29.1 to 65.6) | 44.4 (29.3 to 65) | -0.6 (-6.7 to 6) |
| Turkey | Incidence | 7.6 (6.2 to 9.3) | 7.5 (6.1 to 9.1) | -2.2 (-2.2 to -2.2) |
|  | Prevalence | 292.6 (238.9 to 355) | 293.3 (239.4 to 355.8) | 0.2 (0.2 to 0.2) |
|  | YLDs | 44.7 (29.3 to 65.8) | 44.9 (29.5 to 66.2) | 0.3 (-6.4 to 6.5) |
| United Arab Emirates | Incidence | 7.5 (6.1 to 9.1) | 7.5 (6 to 9) | -0.6 (-0.6 to -0.6) |
|  | Prevalence | 328.5 (269.7 to 397.5) | 331.2 (271.9 to 400.8) | 0.8 (0.7 to 0.9) |
|  | YLDs | 50.2 (32.2 to 73.4) | 50.5 (33 to 73.5) | 0.6 (-6.3 to 7.9) |
| Yemen | Incidence | 7.6 (6.2 to 9.1) | 7.6 (6.3 to 9.2) | 0.6 (0.6 to 0.6) |
|  | Prevalence | 285.9 (234.4 to 346.8) | 286 (234.6 to 347) | 0 (0 to 0.1) |
|  | YLDs | 43.3 (28.6 to 62.4) | 43.5 (28.3 to 62.8) | 0.4 (-6.1 to 6.9) |

Data in parenthesis are 95% Uncertainty Interval (95% UI); YLDs= Years Lived with Disability
